# Dual-emission nitrogen-doped carbon dots for ratiometric and smartphone-based detection of levofloxacin

**DOI:** 10.1039/d5ra05645d

**Published:** 2025-10-21

**Authors:** Sivan A. Abubakr, Sewara J. Mohammed, Azad H. Alshatteri

**Affiliations:** a Department of Chemistry, College of Science, Charmo University 46023 Chamchamal Sulaymaniyah Kurdistan Regional Government Iraq; b Department of Chemistry, College of Science, University of Sulaimani 46001 Sulaymaniyah Kurdistan Regional Government Iraq Sewara.mohammed@univsul.edu.iq; c Department of Chemistry, College of Education, University of Garmian 46021 Kalar Sulaymaniyah Kurdistan Regional Government Iraq azadalshatteri@garmian.edu.krd

## Abstract

Levofloxacin (LEV) is a third-generation fluoroquinolone commonly applied in treatment against infections in both humans and animals. However, its overuse has led to increasing concerns regarding environmental contamination, health risks, and the emergence of antibiotic-resistant bacteria. In this study, we present a novel dual-emission ratiometric fluorescence probe for the detection of LEV, utilizing the intrinsic green fluorescence of LEV as the sensing signal and blue-emissive nitrogen-doped carbon dots (N-CDs) as an internal reference. N-CDs were synthesized *via* a one-pot hydrothermal method using 4-aminoantipyrine (4AA) and hydrazine hydrate (HH) as precursors. Elemental analysis revealed a high nitrogen content (22.1%), which enhanced the photoluminescence properties, achieving a quantum yield of 35.6%. Surface functional groups (–OH, –NH_2_, and –COOH) contributed to excellent water dispersibility. Comprehensive characterization (FTIR, ^1^H-NMR, ^13^C-NMR, and Raman spectroscopy, XPS, XRD, and TEM) was conducted, and a plausible formation mechanism of N-CDs was proposed. Upon interaction with LEV, a clear fluorescence shift from blue to green enabled ratiometric detection, which was further implemented using a smartphone-based sensing platform. The probe exhibited a detection limit of 43 μmol L^−1^, a linear range of 0.4–20 μmol L^−1^, and a rapid response time of 3 min. Successful application to pharmaceutical samples demonstrated the probe's high accuracy and precision.

## Abstract 2

Blue-emissive nitrogen-doped carbon dots enable dual-emission ratiometric and smartphone-based detection of levofloxacin with high sensitivity, rapid response, and excellent selectivity.

## Graphical abstract

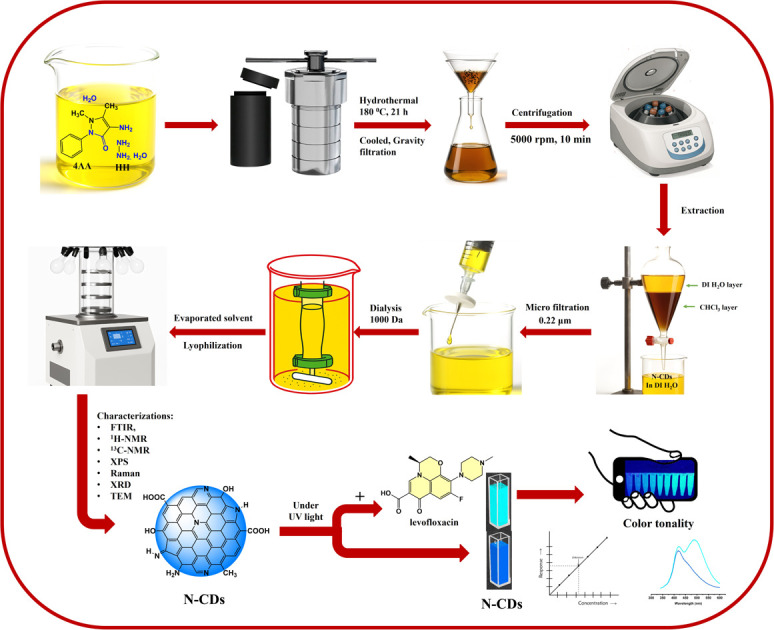

## 1. Introduction

Levofloxacin (LEV) is a broad-spectrum fluoroquinolone antibiotic used universally in human and veterinary medicine and has become a matter of concern due to its increased overuse.^1^ Its common existence in the environmental matrices as well as food products, including meat and milk, leads to the proliferation of antibiotic resistance, environmental contamination, and the subsequent health hazards.^2^

Several methods, such as the electrochemical approach,^3^ high-performance liquid chromatography (HPLC),^4^ capillary electrophoresis,^5^ and chemiluminescence,^6^ have been employed for LEV detection. Although these techniques are sensitive and accurate, they are usually limited by their high operation costs, complex sample preparation, and time-consuming processes. Additionally, they may not be convenient for the quick or on-site detection of LEV, especially in low-resource settings.

An alternative technique that has been proven to be promising is fluorescence (FL) sensing owing to its rapid response, sensitivity, affordability, and ease of operation.^7^ Carbon dots (CDs) represent one of the numerous fluorescent nanomaterials that have attracted significant interest due to their excellent photostability, tunable emission, low toxicity, and compatibility with the environment.^8,9^ The optical properties of CDs can be controlled rather effectively by adjusting their size, surface functionality, and doping with heteroatoms.^10^ CDs derived from small organic molecules provide high levels of control in the incorporation of functional groups and emission properties, which make them very appealing for their application in sensing.^11^

Nitrogen-doped carbon dots (N-CDs) have especially exhibited excellent fluorescence, strong water dispersibility, and low biological toxicity.^12,13^ Their quantum yield, chemical stability, and biocompatibility are improved by nitrogen doping, expanding their application range to bioimaging, photocatalysis, and drug sensing.^14,15^

Despite these advances, most existing fluorescent sensors for LEV rely on single-emission systems, which are highly susceptible to variations in excitation intensity, probe concentration, and environmental conditions. Such fluctuations can compromise analytical accuracy and reproducibility.^16^ Ratiometric fluorescent sensors, which utilize two distinct emission signals for internal calibration, offer a powerful solution to these limitations.^17–19^ These systems improve analytical precision and enable visual detection under UV light, facilitating field-deployable applications.^20^ However, many reported ratiometric systems suffer from complex fabrication procedures, multicomponent designs, or limited stability, which hinder their practical use.^21,22^

This study introduces the synthesis and application of novel dual-emission N-CDs as a ratiometric fluorescent probe for the detection of LEV. N-CDs were prepared *via* a simple one-step hydrothermal method using 4-aminoantipyrine (4AA) and hydrazine hydrate (HH) as precursors. The resulting N-CDs exhibit strong blue emission and enable the ratiometric detection of LEV through the intrinsic green fluorescence of the antibiotic. A clear blue-to-green fluorescence transition under UV light allows for visual detection, while quantitative analysis was achieved using a smartphone-based RGB (red, green, blue) platform. This approach provides a rapid, cost-effective, and accurate strategy for LEV monitoring, with strong potential for applications in pharmaceutical quality control and point-of-care diagnostics.

## 2. Experimental section

### 2.1. Materials

All reagents and chemicals used in this research were of analytical grade or laboratory reagent (LR) grade and were used without further purification. 4-Aminoantipyrine (98%) and chloroform (CHCl_3_) were obtained from Sigma-Aldrich. Hydrazine hydrate (99% LR grade) was purchased from SDFCL. Ethanol was purchased at the Tianjin Fengchuan Chemical Reagent Technology Co., Ltd (Tianjin, China), and levofloxacin was obtained from Pioneer Co. A buffer solution at pH 4.0 was acquired from Biochem. Citric acid, starch, and ferric chloride were purchased from Merck and were used as received.

### 2.2. Instrumentation

Fourier-transform infrared (FT-IR) spectra were obtained using a PerkinElmer spectrophotometer with KBr pellets. Nuclear magnetic resonance spectra, including both ^13^C-NMR and ^1^H-NMR, were obtained using a JEOL DRX-600 MHz spectrometer (JNM-ECZ600R/S1) in D_2_O and CDCl_3_ as solvents. Cary 60 and Cary Eclipse spectrophotometers (Agilent Technologies, USA) were used to obtain ultraviolet-visible (UV-Vis) absorption and photoluminescence (PL) emission spectra, respectively. An X-ray diffractometer (Bruker D8 ADVANCE) was used to carry out X-ray diffraction (XRD). Raman and X-ray photoelectron spectroscopy (XPS) spectra were collected using a Thermo Scientific DXR Raman spectrometer and Thermo Fisher ESCALAB 250Xi XPS, respectively. Transmission electron microscopy (TEM) studies were conducted using an FEI Tecnai G2 F30.

### 2.3. Synthesis of N-CDs

N-CDs were effectively synthesized through a hydrothermal process using 4-aminoantipyrine (4AA) and hydrazine hydrate (HH) as precursors in deionized (DI) water. Initially, 4AA (0.025 mol, 5.081 g) was dissolved in 50 mL of DI water, followed by the addition of HH (0.05 mol, 2.48 mL). The resulting solution was transferred to a Teflon-lined stainless-steel autoclave and subjected to hydrothermal treatment at 180 °C for 21 h. Upon naturally cooling to room temperature, a brown solution was obtained.

**Scheme 1:**
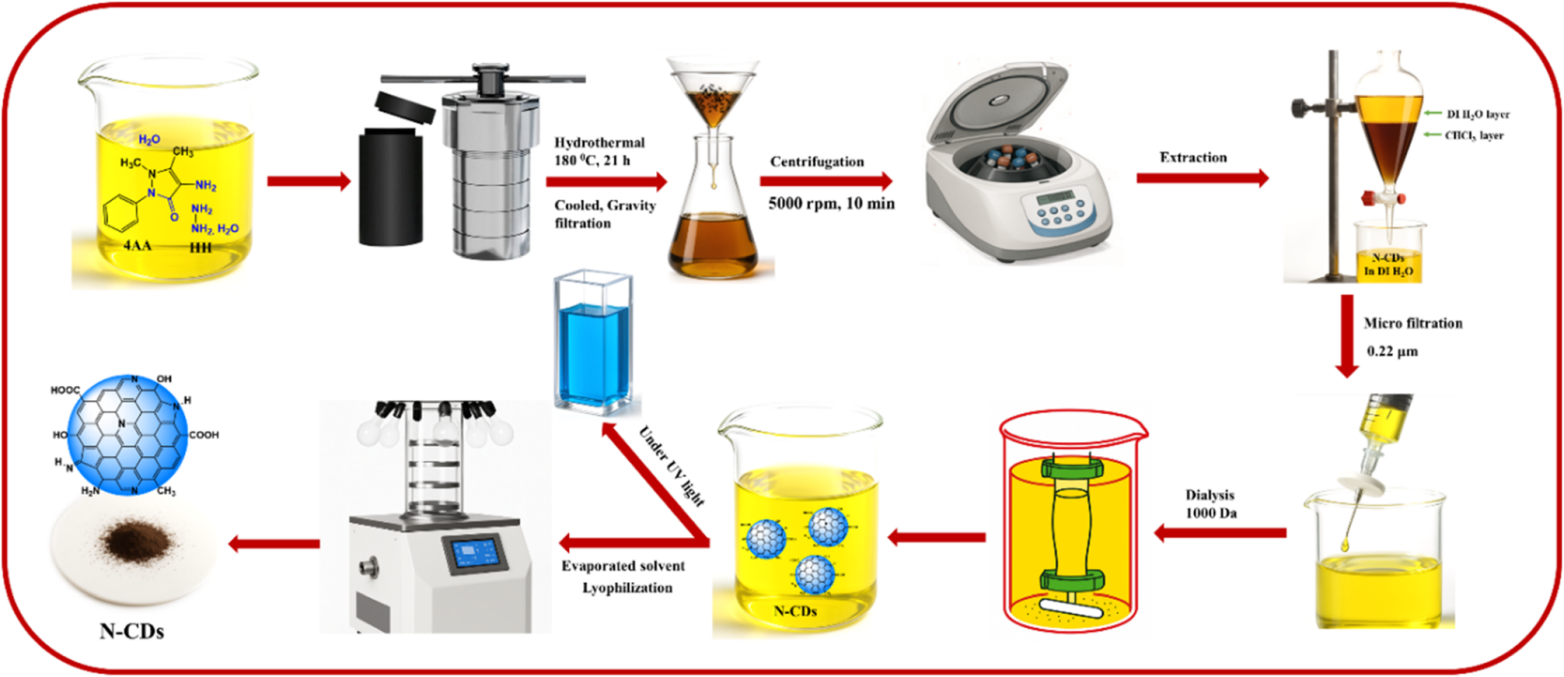
Schematic of the synthesis and purification of N-CDs from 4AA and HH.

The reaction mixture was gravity-filtered twice and then centrifuged at 5000 rpm for 10 min to remove large carbonaceous aggregates. Subsequently, the extraction procedure was performed three times using chloroform as the solvent to eliminate unreacted 4AA, and the resulting aqueous layer was filtered through a 0.22 μm sterile PTFE syringe filter. Further purification involved dialysis using a 1000 Da membrane bag in distilled water to remove residual HH and high-molecular-weight carbon species. The dialysis was carried out with continuous magnetic stirring, replacing the water every 6 h over a 24 h period. Finally, the solvent was removed by lyophilization, yielding dark brown N-CDs, as illustrated in Scheme 1. The product was stored at room temperature for subsequent characterization and applications.

### 2.4. Fluorometric determination of LEV

To prepare the working solution of N-CDs, 5.0 mL of the original N-CDs suspension was transferred into a 100 mL volumetric flask and diluted to the mark with DI water. A LEV stock solution (0.2 mM) was prepared using DI water as the diluent. For fluorescence measurements, 2.0 mL of N-CDs solution was mixed with varying concentrations of LEV, and the total volume was adjusted to 5.0 mL using an optimized acetate buffer solution (pH 7.0).

The mixtures were thoroughly vortexed and incubated at room temperature for 3 min to ensure equilibrium. Fluorescence spectra were then recorded over the 330–600 nm range using an excitation wavelength of 320 nm. The ratiometric fluorescence intensity was determined by calculating the *I*_493_/*I*_418_ ratio, corresponding to the emission peaks of LEV and N-CDs, respectively. Each measurement was conducted in triplicate to ensure reproducibility.

For smartphone-based detection, the prepared solutions were transferred into transparent vials and photographed under 365 nm UV illumination using a smartphone camera placed inside a custom-built dark chamber to eliminate ambient light interference.

To assess the applicability of the developed ratiometric fluorescence method, LEV tablets from three pharmaceutical brands were analyzed. Each tablet (500 mg) was de-coated and ground, and 9 mg of the powder was dissolved in deionized water. The solutions were filtered to remove excipients and diluted to 250 mL.

To evaluate the method's accuracy, the spike recovery approach was employed by adding known concentrations of LEV to the samples. The fluorescence intensity ratio (*I*_493_/*I*_418_) was determined, and recovery percentages were calculated by comparing the experimental values with those predicted by the standard calibration curve, confirming the method's accuracy and reliability.

### 2.5. Smartphone-based determination of LEV

Fluorescence imaging was performed using a smartphone equipped with a high-resolution camera (Huawei P30 Pro). Excitation was provided by a handheld UV lamp emitting at 365 nm. To ensure consistent imaging conditions and minimize ambient light interference, a custom-built dark box was fabricated from black foam board. The enclosure included a dedicated slot for secure smartphone placement and an internal sample holder designed to maintain a fixed distance between the excitation source and the sample vials. Fluorescence intensity was quantified from captured images using Color Grab, a mobile application.

## 3. Results and discussion

### 3.1. Synthesis and spectroscopic characterization of N-CDs

N-CDs were synthesized using a straightforward, green hydrothermal approach using 4AA and HH as precursors, with the reaction carried out at 180 °C for 21 h. To investigate the structural evolution and surface functional groups of the synthesized N-CDs, several analytical techniques, including FTIR spectroscopy, ^1^H-NMR spectroscopy, ^13^C-NMR spectroscopy, Raman spectroscopy, XPS, XRD, and TEM, were employed. These complementary methods provide detailed insights into the chemical transformation of 4AA and HH under hydrothermal conditions.

#### 3.1.1. FT-IR analysis of N-CDs

**Fig. 1:**
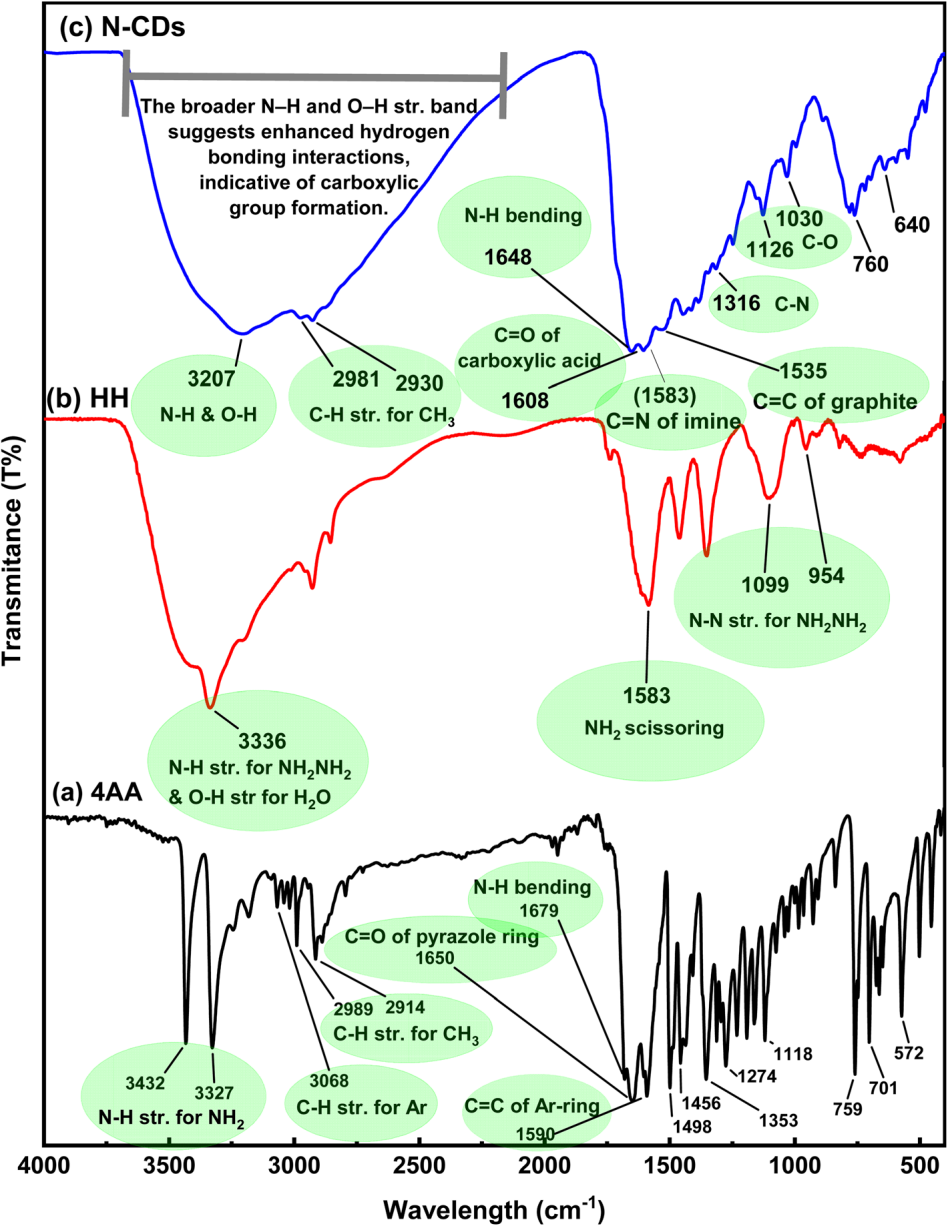
FT-IR spectra of (a) 4AA, (b) HH, and (c) N-CDs.

FTIR spectroscopy was used to analyze the chemical changes and surface functionalities of the synthesized N-CDs compared to their precursors (Fig. 1a–c and Fig. S1a and b, SI).

The FTIR spectrum of 4AA (Fig. 1a, black spectrum) shows strong bands at 3432 and 3327 cm^−1^, corresponding to N–H stretching vibrations of primary amine groups.^23,24^ Aromatic and methyl C–H stretching appears at 3068, 2989, and 2914 cm^−1^.^25^ A sharp peak at 1650 cm^−1^ is assigned to C=O stretching in the pyrazole ring, and the band at 1590 cm^−1^ corresponds to aromatic C=C stretching.^26,27^

The FTIR spectrum of HH (Fig. 1b, red spectrum) exhibits a broad band at 3336 cm^−1^, attributed to overlapping O–H and N–H stretching vibrations, indicating hydrogen bonding.^28^ A peak at 1583 cm^−1^ corresponds to the NH_2_ bending (from NH_2_NH_2_).^29^ Bands at 1099 and 954 cm^−1^ are assigned to N–N stretching and NH_2_ scissoring modes, respectively.^28^

Following hydrothermal treatment, the FTIR spectrum of N-CDs (Fig. 1c, blue spectrum) displayed significant changes. A broad band at 3207 cm^−1^ indicates enhanced hydrogen bonding,^30^ potentially due to overlapping O–H and N–H vibrations.^31^ The broadening of this band, relative to the precursors, suggests surface oxidation and carboxylic acid formation.^32^ The C–H stretching for aliphatic groups was observed at 2981 and 2930 cm^−1.^^33^ Notably, a new peak at 1608 cm^−1^ was attributed to the C=O stretching vibration of carboxylic acids,^34,35^ while a distinct band at 1648 cm^−1^ corresponds to N–H bending vibrations.^36,37^ The presence of an imine-related band at 1583 cm^−1^^38^ supports the formation of C=N linkages during condensation reactions. Furthermore, the appearance of bands at 1535 cm^−1^ (C=C of graphitic domains),^39^ 1316 cm^−1^ (C–N stretching),^40,41^ and 1030/1126 cm^−1^ (C–O stretching) confirmed the successful incorporation of nitrogen- and oxygen-containing surface functionalities.^42,43^

These findings indicate that the resulting N-CDs are enriched with polar functional groups (–COOH, –OH, –NH_2_, and –C=N) that enhance their hydrophilicity and chemical reactivity.

#### 3.1.2. ^1^H-NMR analysis of N-CDs

**Fig. 2:**
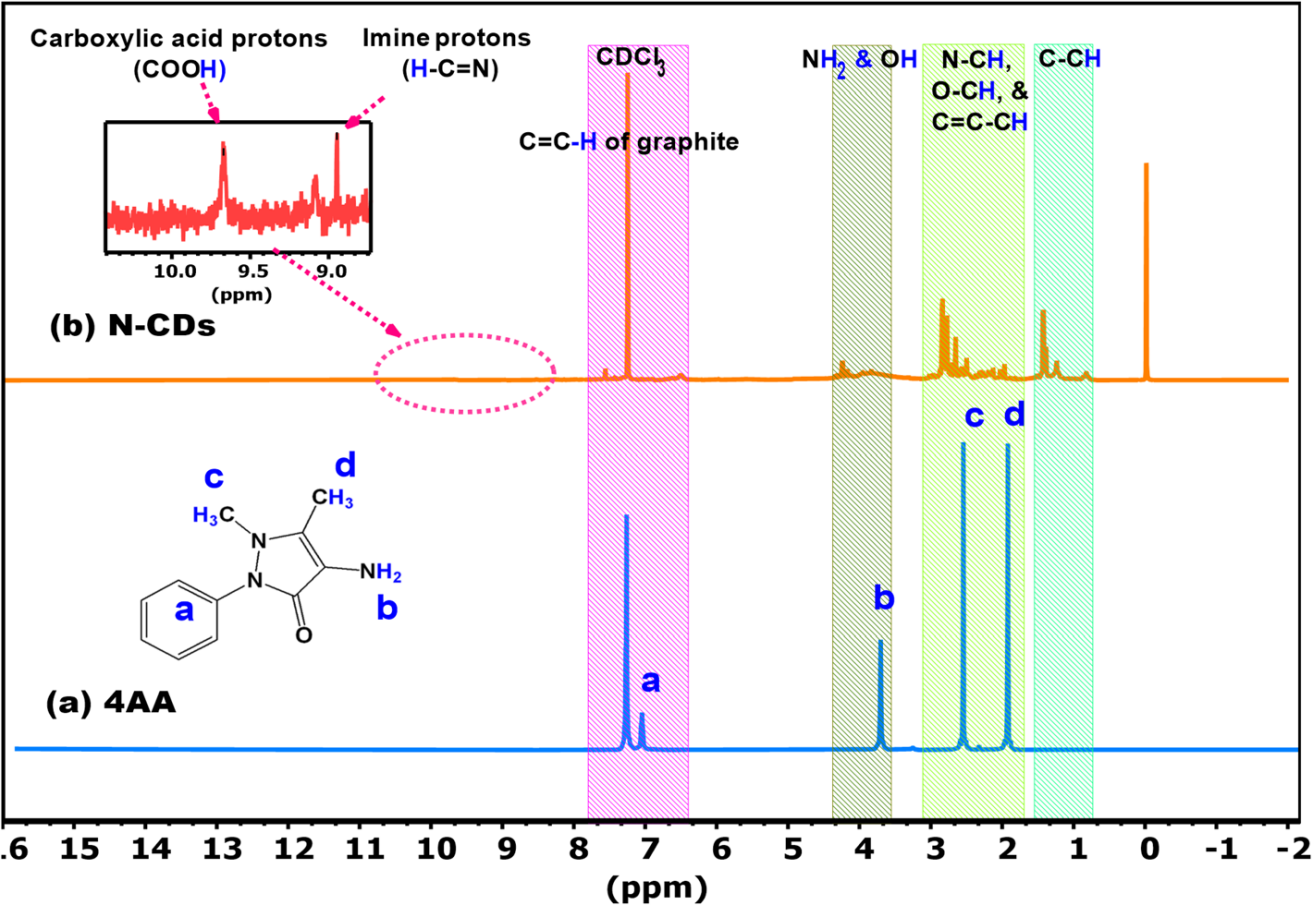
^1^H-NMR spectra of (a) 4AA and (b) N-CDs recorded in CDCl_3_.

^1^H-NMR spectroscopy was employed to track the structural transformation of the precursors into N-CDs. The spectra of 4AA and N-CDs, recorded in CDCl_3_, show notable shifts and signal broadening, indicating structural evolution, as shown in Fig. 2a and b and Fig. S2a and b (SI).

The ^1^H-NMR spectrum of 4AA (Fig. 2a, blue spectrum) displays four distinct signals (a–d), corresponding to four chemically distinct proton environments. Aromatic protons (signal a) appear as multiplets in the 7.5–7.0 ppm region, consistent with a phenyl ring.^44^ Amino protons (signal b) appear at 3.9 ppm.^45^ Two methyl groups (signals c and d) on the pyrazole ring are observed at 2.3 and 1.9 ppm, respectively.^24^

After hydrothermal treatment, the ^1^H-NMR spectrum of N-CDs (Fig. 2b, red spectrum) showed broad, overlapping signals typical of disordered structures with diverse surface functionalities. Signals in the 10.2–9.0 ppm range are attributed to carboxylic acid (–COOH) and imine (–CH = N–) protons.^46^ Peaks between 7.5–6.0 ppm suggest the presence of sp^2^-hybridized aromatic protons from graphitic domains.^47^ Signals between 4.5–4.0 ppm arise from hydroxyl and amino groups,^48^ while peaks in the 3.0–1.0 ppm region are attributed to aliphatic and heteroatom-bound protons (*e.g.*, N–CH, O–CH, and C–C=CH).^49^

These findings corroborate the successful conversion of 4AA and HH into N-CDs bearing diverse functional groups, aligning well with the FTIR findings.

#### 3.1.3. ^13^C-NMR analysis of N-CDs

**Fig. 3:**
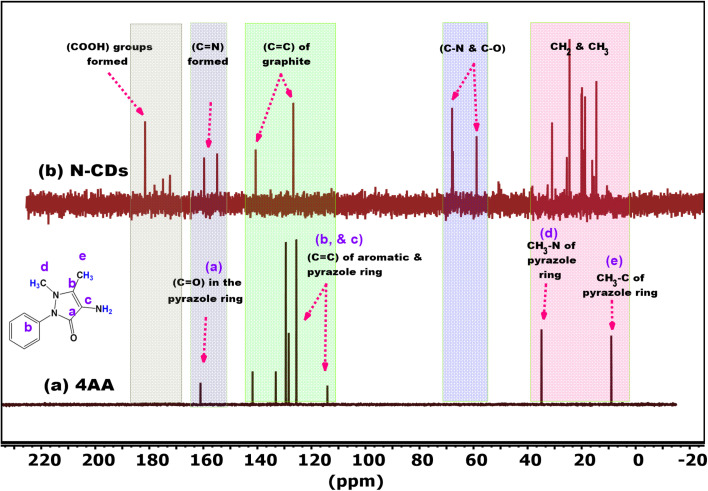
^13^C-NMR spectra of (a) 4AA and (b) N-CDs recorded in D_2_O.

^13^C-NMR spectroscopy was performed to elucidate further the carbon environments in 4AA and the synthesized N-CDs. The spectra, recorded in D_2_O, are presented in Fig. 3a and b and Fig. S3a and b (SI).

The ^13^C-NMR spectrum of 4AA (Fig. 3a, black spectrum) displays five distinct signals (a–e), consistent with its molecular structure. The carbonyl carbon (signal a) of the pyrazole ring appears at 161 ppm.^50^ Aromatic and pyrazole ring carbons (signals b and c) appear in the 141–114 ppm range, representing sp^2^-hybridized carbons.^51^ The methyl carbon attached to the nitrogen (signal d) is observed at 34 ppm, while the other methyl group (signal) appears at 9 ppm^52^.

In contrast, the ^13^C-NMR spectrum of N-CDs (Fig. 3b, black spectrum) shows broad, overlapping signals, indicating a highly disordered and functionalized carbon framework. Signals in the 181–172 ppm range are assigned to carboxylic acid groups (–COOH), suggesting oxidative surface modification.^53^ Peaks between 159–154 ppm correspond to imine (C=N) groups formed through dehydration and condensation.^54^ Signals in the 140–126 ppm region signify sp^2^-hybridized carbons within graphitic structures.^55^ The 67–58 ppm region includes peaks from C–N and C–O bonds, indicative of nitrogen doping and oxygen-containing functional groups.^24^ Finally, signals below 40 ppm are attributed to aliphatic (CH_2_/CH_3_) carbons, possibly from newly introduced alkyl groups.^56^

These observations provide strong evidence for the successful conversion of 4AA into N-CDs enriched with diverse functional groups.

#### 3.1.4. X-ray photoelectron spectroscopy analysis of N-CDs

**Fig. 4:**
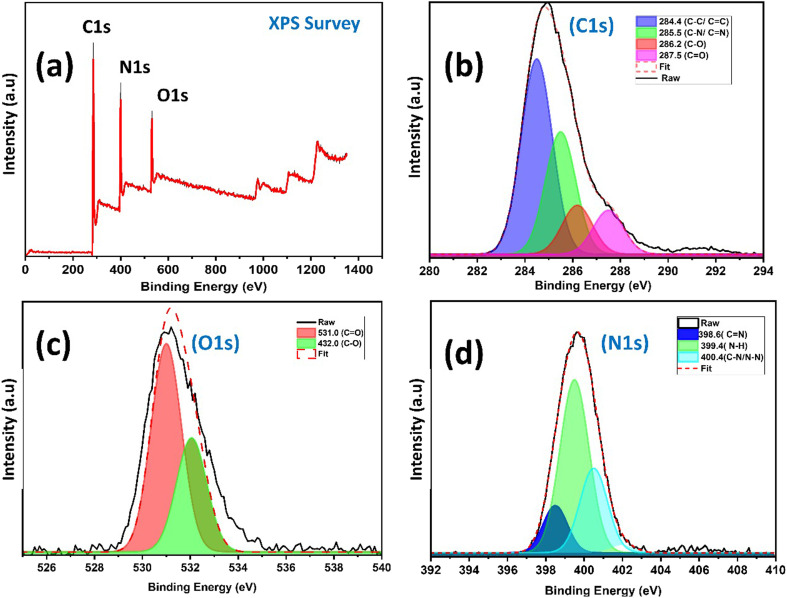
XPS analysis of N-CDs: (a) full survey spectrum and (b) C 1s, (c) O 1s, and (d) N 1s high-resolution spectra.

XPS was employed to analyze the elemental composition and chemical states of the synthesized N-CDs. The XPS survey spectrum confirmed the successful formation of N-CDs, showing the presence of carbon (C 1s: 67.9%), nitrogen (N 1s: 22.1%), and oxygen (O 1s: 10.0%) (Fig. 4a). The corresponding binding energies were observed at 285.0 eV (C 1s), 399.7 eV (N 1s), and 531.2 eV (O 1s).^57^ High-resolution XPS spectra provided further insight into the chemical bonding environments.

The C 1s spectrum (Fig. 4b) was deconvoluted into five components: C–C/C=C (284.4 eV)^58^,^59^, C–N/C=N (285.1 eV),^60^ C–O (286.2 eV),^61,62^ C=O (287.5 eV),^63^ and HO–C=O (289.1 eV).^64^ The O 1s spectrum (Fig. 4c) showed peaks for C=O (531.1 eV), HO–C=O (532.1 eV), C–O (533.5 eV), and O–H (533.8 eV).^41,58^ The N 1s spectrum (Fig. 4d) exhibited peaks corresponding to pyridinic N (C=N, 398.6 eV),^60^ N–H (399.4 eV),^65^ and amide C–N (400.4 eV).^60^

To quantitatively correlate the surface chemistry with the material's properties, the relative abundance of key functional groups was calculated from the deconvoluted high-resolution spectra and is summarized in Table 1. The calculation details, including the use of relative sensitivity factors and the method for converting high-resolution peak areas to whole-surface atomic percentages, are provided in the SI (5. Quantitative XPS Analysis, Table S1). The surface is rich in amine/pyrrolic N–H groups (12.83%), which, along with the significant presence of oxygen-containing groups like carboxyl (–COOH, 1.76%) and hydroxyl (–OH, 0.48%), is crucial for the high aqueous dispersibility of N-CDs and their subsequent interaction with levofloxacin. These XPS findings are consistent with the FTIR, ^1^H-NMR, and ^13^C-NMR spectroscopy results, collectively confirming the functionalized surface of N-CDs.

**Table 1:** Surface functional-group composition of N-CDs obtained from XPS deconvolution

Functional group	% of surface	Origin (core level)	Note
–COOH (carboxyl)	1.76	C 1s (O–C=O)	Confirmed by O 1s stoichiometry
–OH (hydroxyl)	0.48	O 1s (O–H)	May include physisorbed H_2_O
N–H (amine/pyrrolic)	12.83	N 1s (N–H)	–NH_2_ included; XPS cannot isolate the primary amine
–C=N (imine/pyridinic N)	2.91	N 1s (C=N)	

#### 3.1.5. Raman spectroscopy, XRD, and TEM analysis of N-CDs

**Fig. 5:**
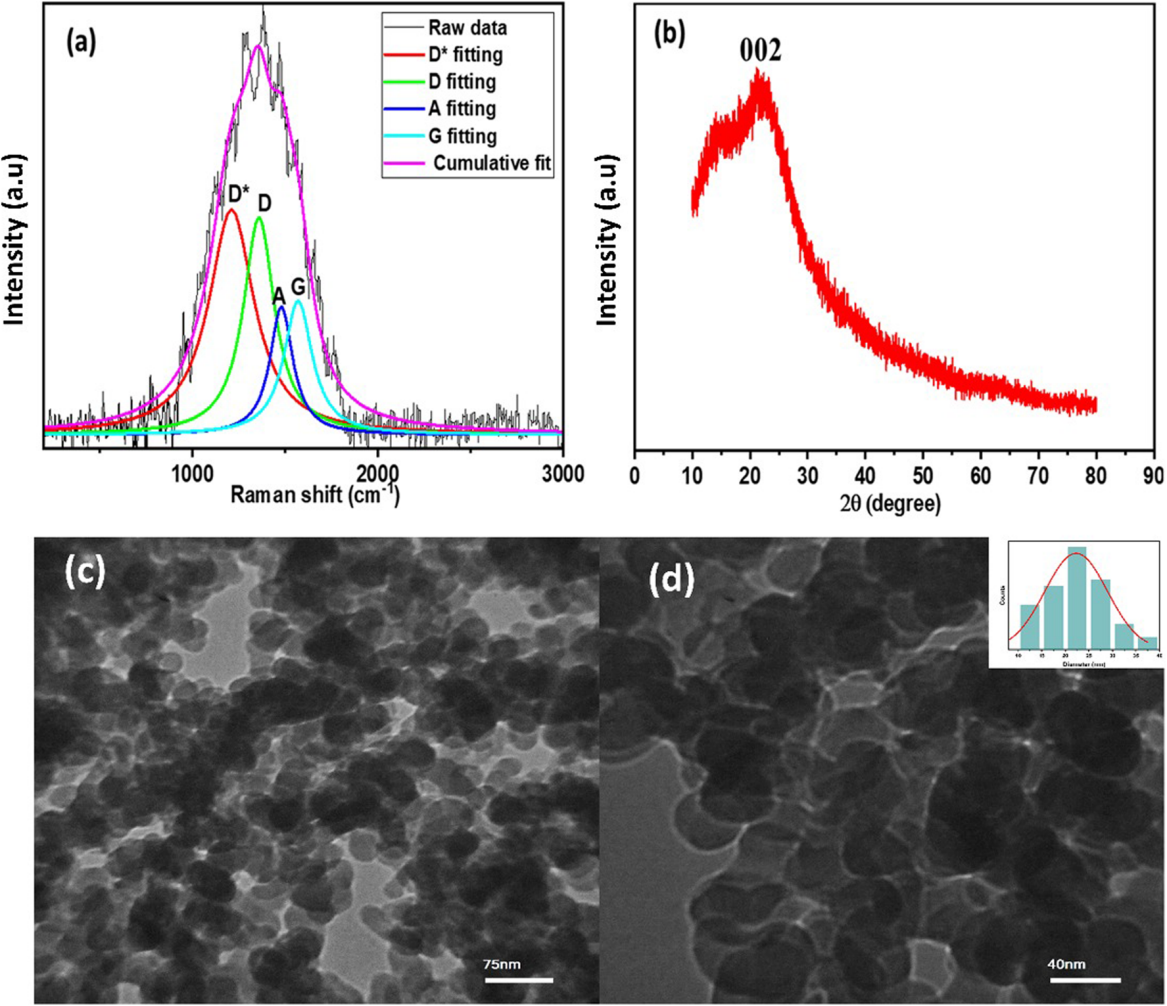
(a) Raman spectrum, (b) X-ray diffraction (XRD) pattern, (c) low-magnification transmission electron microscopy (TEM) image (scale bar: 75 nm), and (d) high-magnification TEM image (scale bar: 40 nm) of the synthesized N-CDs. The inset in (d) shows the particle size distribution histogram derived from the TEM results.

Raman spectroscopy was employed to investigate the structural properties of the synthesized N-CDs (Fig. 5a). The deconvoluted spectrum revealed four distinct peaks at approximately 1210 cm^−1^ (D* band), 1359 cm^−1^ (D band), 1480 cm^−1^ (A band), and 1570 cm^−1^ (G band).^66^ The pronounced D* and D bands suggest a high density of defects and functionalized carbon structures, while the G band confirms the presence of graphitic sp^2^ domains.^67^ The broad nature of the peaks indicates that N-CDs are predominantly amorphous with limited graphitic order. Furthermore, the relatively high *I*_D_/*I*_G_ intensity ratio further supports the presence of structural disorder and oxygen-containing functional groups in the synthesized material.^68^

Powder X-ray diffraction (XRD) analysis of N-CDs (Fig. 5b) shows a broad peak centered around 2*θ* ≈ 20°–25°.^69^ This broad hump reveals their amorphous, graphitic-like structure with a typical interlayer (*d*-spacing) in the range of ∼0.34–0.44 nm, which matches graphitic layers (002 planes) seen in carbon-based nanostructures.^70,71^ This feature is characteristic of a turbostratic structure with disordered graphitic domains.^72^ The broadness of the peak suggests a lack of long-range crystalline order, a typical attribute of carbon dots synthesized *via* chemical methods.^73^ The absence of sharp diffraction peaks further confirmed the amorphous nature of N-CDs, indicating that they consist of randomly stacked graphene-like layers rather than well-ordered graphite.^74^ Similar broad peaks have been reported in previous studies and attributed to sp^2^-hybridized carbon clusters embedded in an sp^3^ matrix.^75^ Additionally, no significant peaks near 44° (2*θ*), which would correspond to the (100) plane of crystalline graphite, were observed, confirming the absence of highly ordered graphitic domains or crystalline impurities.^76^

According to transmission electron microscopy (TEM) analysis (Fig. 5c), N-CDs exhibit a predominantly spherical morphology with sizes ranging from 10 to 40 nm (Fig. 5d). The particles show moderate aggregation and variations in contrast, consistent with the isotropic growth typically observed in carbon dots (CDs) synthesized *via* hydrothermal or solvothermal methods. Such routes often promote clustering due to interactions between surface functional groups.^77^ The observed morphological diversity, including localized electron density variations and irregular shapes, may arise from nitrogen doping, which likely alters nucleation and growth kinetics.^78^ The presence of darker regions in the TEM images suggests that nitrogen incorporation occurs either during or shortly after the formation of the carbon matrix.^61^

#### 3.1.6. Optical properties of N-CDs

**Fig. 6:**
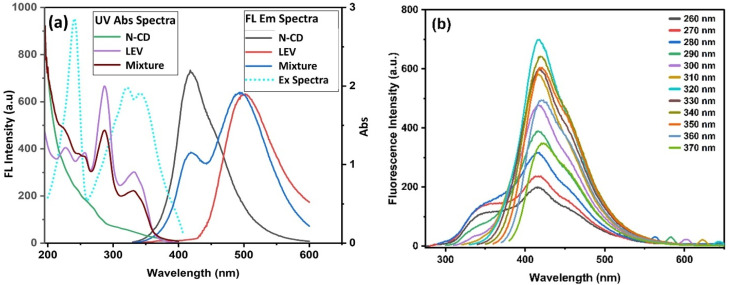
(a) UV-Vis absorption and fluorescence emission spectra of N-CDs, LEV, and their mixture. (b) Excitation-dependent emission spectra of N-CDs at various excitation wavelengths (260–370 nm). The quantum yield (QY%) was determined using fluorescein as a reference standard,^66^ resulting in a value of 36.5%. This value is relatively high and exceeds that of several other reported CDs used as LEV nanoprobes, as shown in

The optical properties of the synthesized N-CDs, levofloxacin (LEV), and their mixture were systematically investigated. The UV-Vis absorption spectra (Fig. 6a) show that the N-CDs/LEV mixture exhibits the combined absorption features of both individual components, confirming the coexistence of both species in solution without the formation of a new ground-state complex.

The fluorescence emission spectra provided further insight. N-CDs exhibited strong blue emission at 418 nm. Notably, while LEV alone showed weak intrinsic fluorescence under these conditions, its emission at ∼493 nm was significantly enhanced in the mixture upon excitation at 320 nm. This key observation indicates that N-CDs create Table 2.an optimal local environment that enhances the intrinsic fluorescence of LEV, rather than engaging in a quenching-based interaction.^57^ The presence of two distinct, well-resolved peaks from N-CDs (418 nm) and LEV (493 nm) formed the ideal basis for the development of a ratiometric probe.

**Table 2:** Comparison of the quantum yields and emission wavelengths of various reported carbon dots with those of N-CDs

Type of carbon dot	Emission color	QY	Ref.
DE-P-CDs	Red	4.2%	17
CDs	Near IR	0.2%	66
CDs	Black	9.5%	80
ONCDs	Green	10%	52
CDs	Yellow	17.4%	60
N-CDs	Blue	36.5%	Current work

N-CDs themselves exhibited excitation-dependent fluorescence behavior across the 260–370 nm range (Fig. 6b). This effect is attributed to the intrinsic structural heterogeneity of N-CDs, including a distribution of particle sizes and diverse surface functional groups, which create multiple emissive traps and energy states. The optimal excitation and emission wavelengths for N-CDs were determined to be 320 nm and 418 nm, respectively (Fig. 6b).

The influence of pH on the N-CD–LEV system was also evaluated (Fig. S4). The fluorescence intensity was found to be strongly pH-dependent. The system performed optimally at pH 4, where minimal fluorescence quenching was observed, and the two characteristic emission peaks (418 nm for N-CDs and 493 nm for LEV) were most distinct and stable.^79^ This pH was therefore selected for all subsequent sensing experiments.

### 3.2. Proposed mechanism of N-CDs formation

**Scheme 2:**
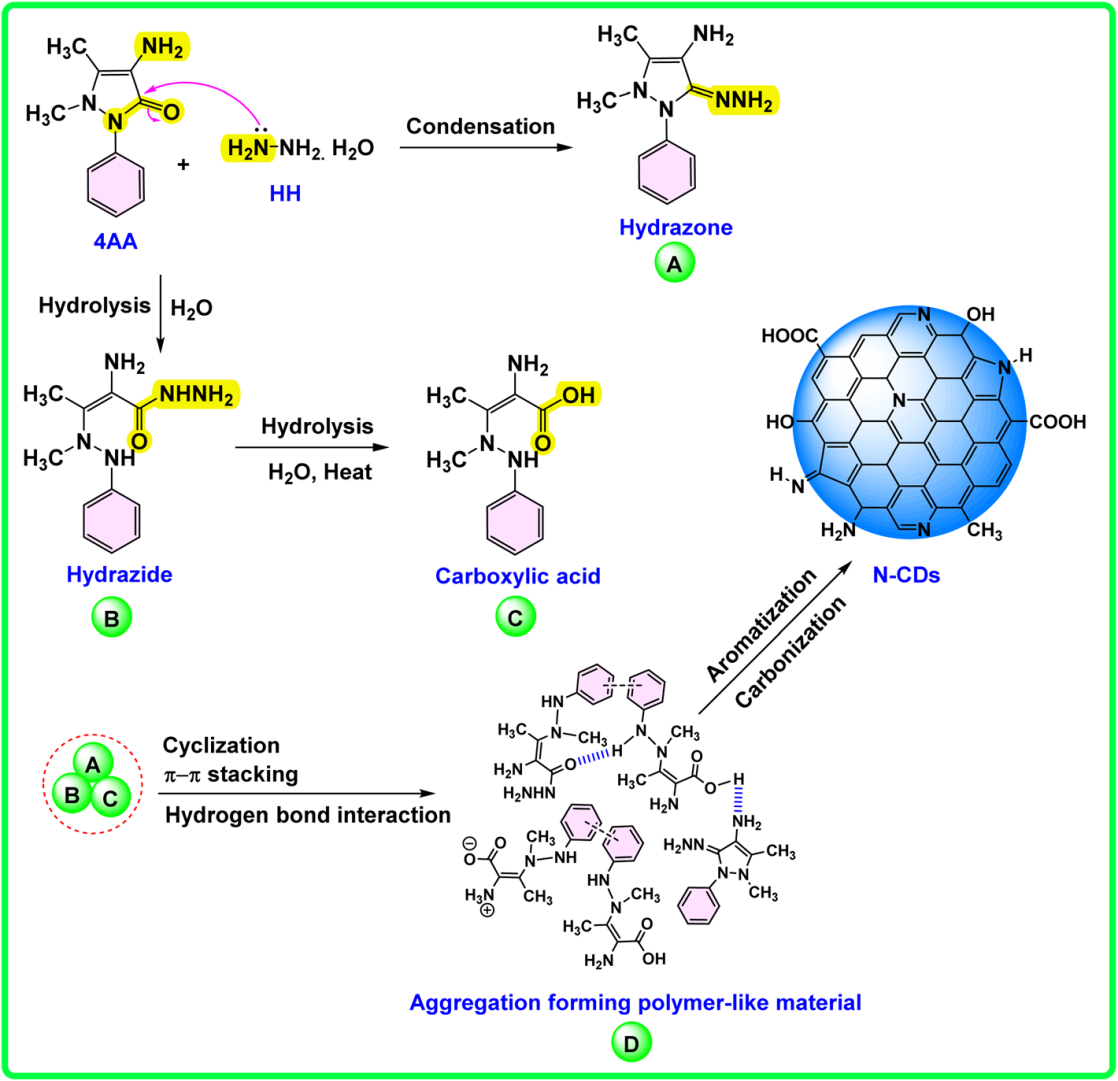
Proposed mechanism of the formation of N-CDs from 4AA and HH under hydrothermal conditions.

Although the precise mechanism of N-CDs formation remains under investigation, a plausible pathway is proposed based on spectroscopic analyses and previous literature, as illustrated in Scheme 2. Initially, 4AA undergoes condensation with HH to form a hydrazone intermediate (A) containing an imine (C=N) linkage.^81^ Simultaneously, partial hydrolysis of the amide group in 4AA yields a hydrazide intermediate (B), which can further hydrolyze under aqueous and thermal conditions to generate a carboxylic acid derivative (C).^82,83^ These intermediates (A–C) undergo cyclization, dehydration, π–π stacking, and hydrogen bonding interactions, leading to a polymer-like aggregated structure (D).^84^ This assembly facilitates aromatization and carbonization, producing a nitrogen-doped graphitic carbon core.^85^ Subsequent surface oxidation and fragmentation introduce functional groups such as –COOH, –OH, and –NH_2_, which enhance the hydrophilicity and chemical reactivity of the resulting N-CDs.^82^

The resulting N-CDs are rich in amino, imine, hydroxyl, and carboxyl groups, imparting water solubility and chemical versatility.

### 3.3. Determination of LEV

#### 3.3.1. Ratiometric fluorimetry

**Fig. 7:**
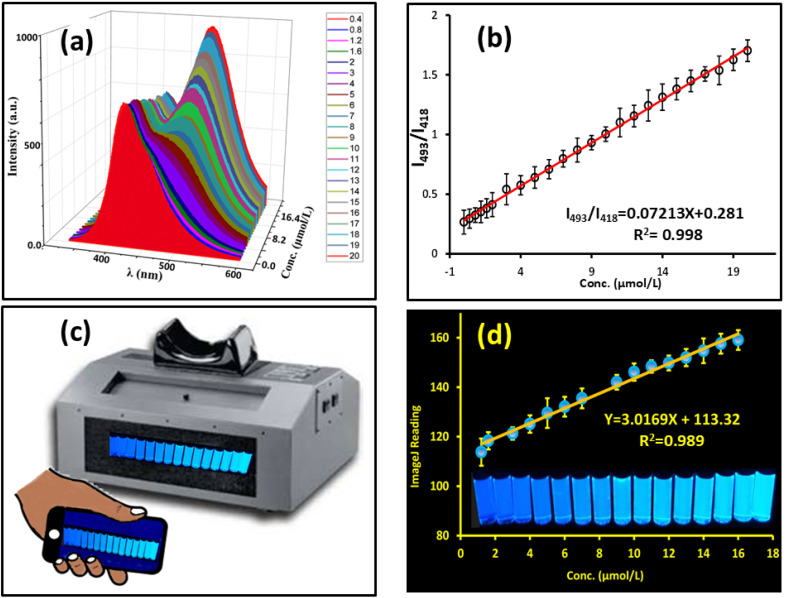
(a) Fluorescence spectra of N-CDs with varying LEV concentrations. (b) Linear relationship between the N-CD fluorescence intensity ratio (*I*_493_/*I*_418_) and LEV concentration. (c) Schematic of the smartphone-assisted platform for capturing and recognizing the fluorescent colors. (d) Smartphone readings *versus* LEV concentrations. Inset: corresponding photographs of the fluorescent solutions under UV light, showing a gradual color change with increasing LEV concentration.

Ratiometric fluorescence sensing is a robust strategy for improving the accuracy and reproducibility of fluorescent probes by incorporating an internal reference signal.^86^ In this study, blue-emissive N-CDs with a stable emission at 418 nm were employed as the reference fluorophore. Upon interaction with LEV, a distinct green emission peak emerges at 493 nm, attributable to the intrinsic fluorescence of LEV (Fig. 7a). While the emission at 418 nm remains largely unchanged, the appearance and growth of the 493 nm peak enable ratiometric detection based on the intensity ratio *I*_493_/*I*_418_.

A linear correlation was observed between *I*_493_/*I*_418_ and LEV concentrations over the range of 0.40–20.00 μmol L^−1^ (Fig. 7b). The method confirmed a detection limit (LOD) of 43 nmol L^−1^, with excellent linearity (*R*^2^ = 0.998).

#### 3.3.2. Smartphone-based detection

By using a smartphone-enabled sensing platform, we have revealed real-time visual sensing and quantification of levofloxacin (LEV) using fluorescence detection. When excited at 365 nm (ultraviolet (UV) wavelength), the color tonality was shown to change from blue to green, thereby allowing both qualitative visual detection (Fig. 7c) and quantitative determination of concentration.

When the captured fluorescent images were analyzed using the Color Grab smartphone application (version 3.9.2, 2021 Loomatix Ltd), numerical values indicating LEV concentrations were derived, as shown in Fig. 7d. The data were statistically analysed to determine a strong linear correlation between fluorescence intensity and the concentration of LEV in the 0–200 mol L^−1^ range, with the calibration equation *y* = 3.0169 *x* + 113.32, and the coefficient of correlation *R*^2^ = 0.989. The limit of detection (LOD) calculated using the 3-sigma criterion was determined to be 0.98 mmol L^−1^.

These results reveal that the N-CD-based probe effectively meets the requirements for visual detection of LEV, suggesting encouraging opportunities for further development. Table 3 lists several advanced analytical techniques used for LEV detection. In terms of LOD and linear response range, our suggested work is like the alternative approaches.

**Table 3:** Comparative evaluation of the previously reported analytical techniques for quantification with the ratiometric fluorometry and smartphone-based detection of LEV reported in the current study

Method	Linear range (μg mL^−1^)	LOD (μg mL^−1^)	Ref.
UV/Vis derivative spectrophotometric	2–20	0.01	87
Reversed-phase HPLC-UV	4.8–29.04	0.667	88
HPLC-fluorescence	2.5–500 × 10^−3^	0.63 × 10^−3^	
HPLC–MS/MS	0.3–15	0.06	89
Spectrofluorimetric	0.20–3.0	0.10	90
Ultra-performance liquid chromatography	0.5–80	0.10	91
Ratiometric fluorometry	0.14–7.2	0.015	Current work
Smartphone-based detection	0.0–7.20	0.354	Current work

#### 3.3.3. Detection of LEV in real samples and selectivity assessment

To evaluate the practicality of the proposed assay, it was applied to the analysis of three commercially available LEV film-coated tablets. For a comprehensive comparison, spike recovery experiments were evaluated using both the ratiometric fluorometric method (Table S2) and the smartphone-based platform (Table S3). The ratiometric method provided recoveries ranging from 85.0%–100.5%, while the smartphone-based platform achieved values between 84.6%–94.1%. These results demonstrate that the smartphone platform offers a recovery performance comparable to established fluorometry, underscoring its potential for practical, low-cost, and portable analysis.

**Fig. 8:**
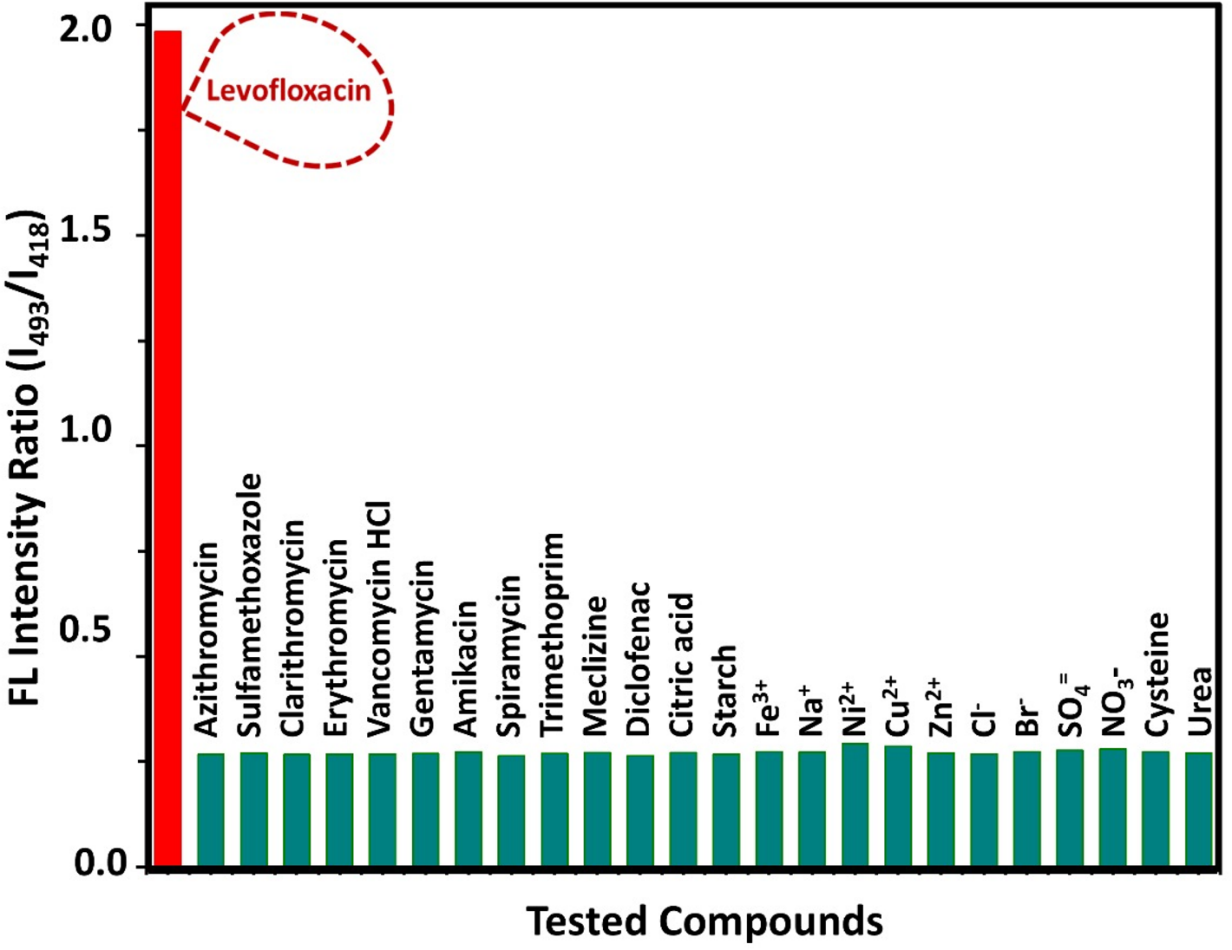
Selectivity assessment of the proposed assay for levofloxacin detection. The analytical signal was recorded for levofloxacin (highlighted in red) and compared with those of a range of commonly used antibiotics (*e.g.*, azithromycin, erythromycin, and ciprofloxacin), pharmaceutical compounds (*e.g.*, diclofenac and meclizine), common tablet excipients (citric acid and starch), common cations (*e.g.*, Fe^3+^ and Na^+^), common anions (*e.g.*, Cl^−^ and SO_4_^2−^) and other biological molecules (*e.g.*, cysteine and urea).

The selectivity of the sensor was rigorously examined against common excipients, co-prescribed drugs (*e.g.*, paracetamol), co-formulated drugs (*e.g.*, fluorometholone), other pharmaceuticals (*e.g.*, diclofenac sodium and etodolac), and various potential interfering ions and biological molecules. As shown in Fig. 8, no significant interference was observed, confirming the high selectivity of the assay towards levofloxacin.

The high selectivity observed can be attributed to the unique design of the sensing system. It is critical to note that N-CDs function exclusively as a stable internal reference fluorophore, exhibiting a constant blue emission at 418 nm that is unaffected by the presence of analytes. The ratiometric response is generated not by modulation of the N-CD fluorescence, but by the inherent green fluorescence of levofloxacin (LEV) itself at ∼493 nm. The quinolone moiety in LEV contains a rigid, polycyclic π-conjugated system that acts as a strong intrinsic fluorophore under the applied experimental conditions. Most of the potentially interfering substances lack a comparable chromophore and thus do not produce significant emission in this specific green spectral region. Therefore, the selectivity arises from the distinct photophysical signature of LEV, which is selectively reported against the stable background of the N-CD emission. This mechanism allows for the specific discrimination of LEV without relying on direct chemical binding interactions with the carbon dots.

## 4. Conclusion

In this study, a novel ratiometric fluorescence probe based on blue-emissive N-CDs was developed for the sensitive and selective detection of LEV. N-CDs, prepared through a one-pot hydrothermal method using 4AA and HH, demonstrated a high quantum yield (36.5%) and excellent water solubility due to their abundant surface functional groups. Comprehensive characterization, including FTIR spectroscopy, ^1^H-NMR spectroscopy, ^13^C-NMR spectroscopy, XPS, Raman spectroscopy, XRD, and TEM, confirmed the successful synthesis and structural properties of N-CDs. The incorporation of LEV induced a green-emissive response, enabling dual-emission ratiometric sensing with a low detection limit of 43 nmol L^−1^ and a wide linear range. A smartphone-based detection platform was successfully employed for visual and quantitative analysis, providing a cost-effective and portable alternative for pharmaceutical quality control. The proposed probe demonstrated high accuracy, selectivity, and recovery in real pharmaceutical samples, positioning it as a promising candidate for point-of-care diagnostics and environmental monitoring of antibiotic residues.

## Conflicts of interest

There are no conflicts of interest to declare

## Supplementary Material

RA-015-D5RA05645D-s001

## Data Availability

All experimental and characterization data are available within the article and its supplementary information (SI). Supplementary information: supplementary Fig. S1–S4 and Table S1, which include comparative FT-IR, ^1^H-NMR, and ^13^C-NMR spectra of the precursor and the synthesized N-CDs, data on the fluorescence intensity of the N-CDs at different pH levels, and detailed results for the determination and recovery of LEV in commercial tablets. See DOI: https://doi.org/10.1039/d5ra05645d.
