# Supplementary material for: Dual-emission nitrogen-doped carbon dots for ratiometric and smartphone-based detection of levofloxacin
Source: RSC Adv. 2025 Oct 21;15(47):40043–55. doi: 10.1039/d5ra05645d (PMC12538674; doi:10.1039/d5ra05645d)
Supplement: RA-015-D5RA05645D-s001 [file RA-015-D5RA05645D-s001.pdf]

Supplementary Information (SI) for RSC Advances.
This journal is © The Royal Society of Chemistry 2025

Electronic Supplementary Information

Dual-Emission Nitrogen-Doped Carbon Dots for Ratiometric and Smartphone-Based Detection of Levofloxacin

Sivan A. Abubakr [a], Sewara J. Mohammed [*b], Azad H. Alshatteri [*c]

a. Department of Chemistry, College of Science, Charmo University, 46023 Chamchamal, Sulaymaniyah, Kurdistan Regional Government, Iraq.
b. Department of Chemistry, College of Science, University of Sulaimani, 46001 Sulaymaniyah, Kurdistan Regional Government, Iraq.
c. Department of Chemistry, College of Education, University of Garmian, 46021 Kalar, Sulaymaniyah, Kurdistan Regional Government, Iraq.

*Corresponding author. E-mail: Sewara.mohammed@univsul.edu.iq: azadalshatteri@garmian.edu.krd

1. **Fig.S1** FT-IR spectra of **(a)** 4AA, and **(b)** N-CDs.
2. **Fig. S2** ^{1}H-NMR spectra of **(a)** 4AA, and **(b)** N-CDs.
3. **Fig. S3** ^{13}C-NMR spectra of **(a)** 4AA, and **(b)** N-CDs.
4. **Fig. S4** The fluorescence intensity of N-CDs at various pH solutions 3-12 with various buffer solutions.
5. **Quantitative XPS Analysis (Table S1.** Elemental composition of the N-CDs obtained from XPS survey spectra.**)**
6. **Table. S2** Determination and recovery of LEV in three commercial LEV tablets.
7. **Table. S3** Determination and recovery of LEV in three commercial LEV tablets using a smartphone-based platform method.

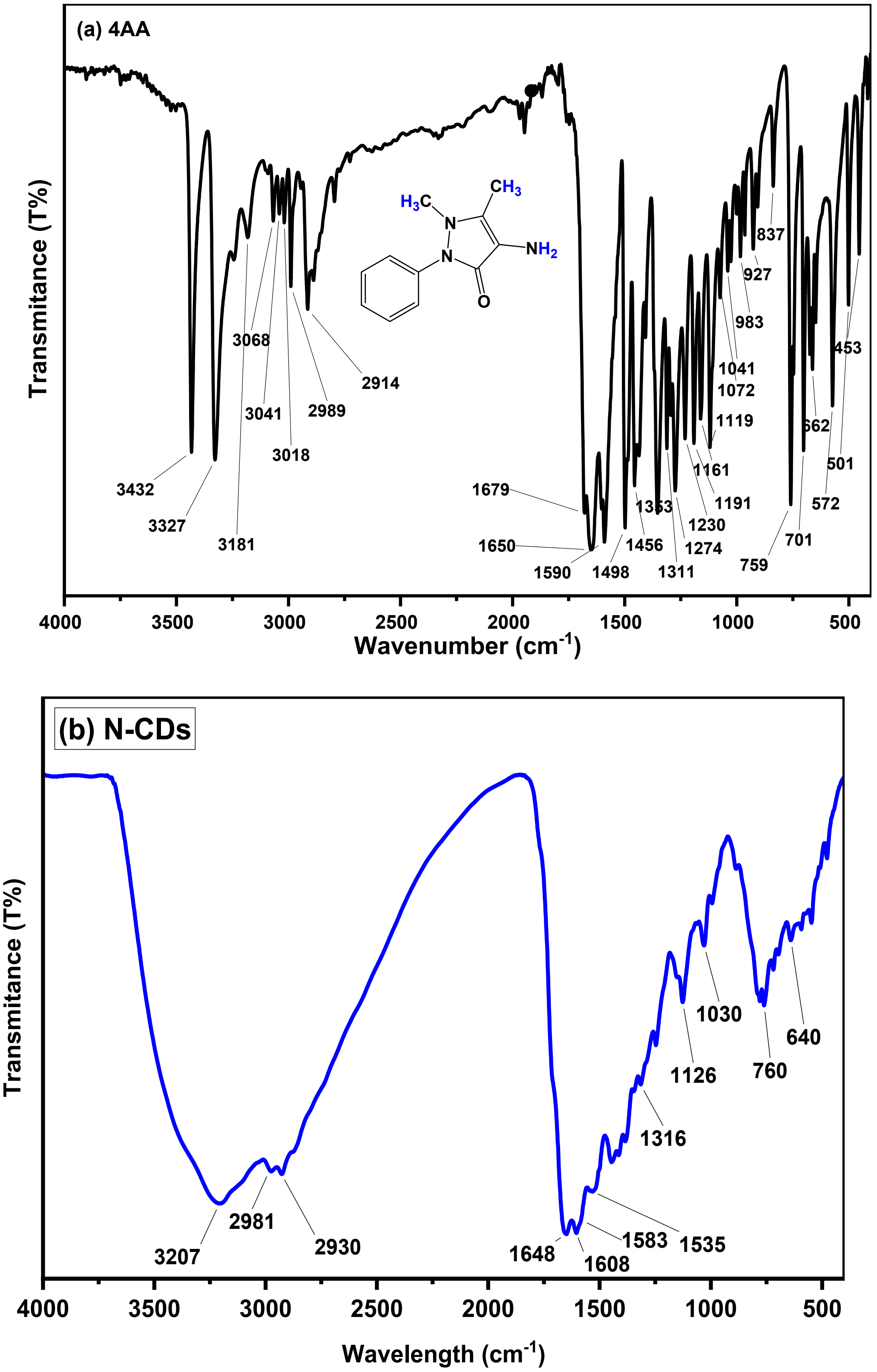

Fig. S1 FT-IR spectra of **(a)** 4AA, and **(b)** N-CDs.

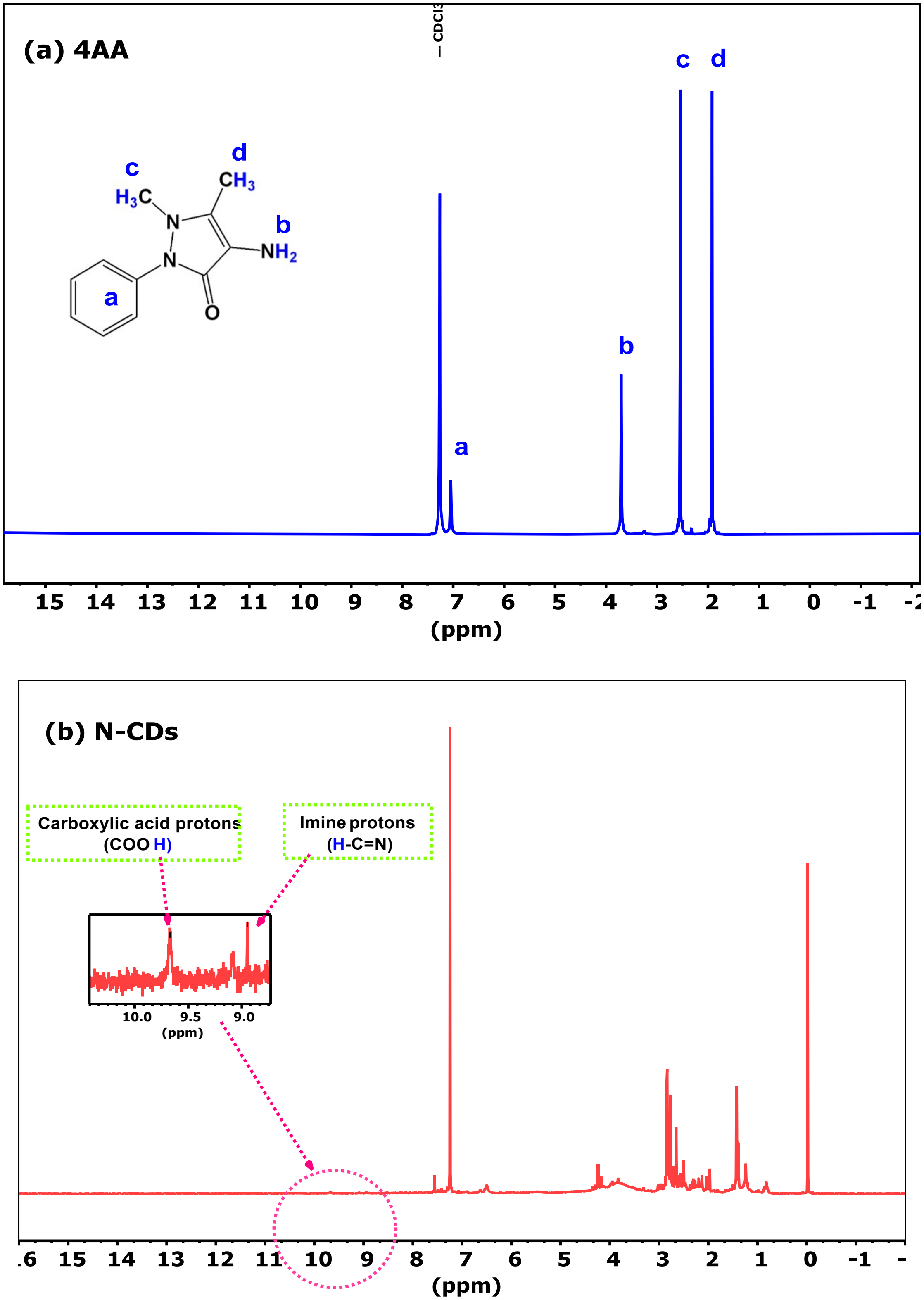

Fig. S2 ^{1}H-NMR spectra of **(a)** 4AA, and **(b)** N-CDs.

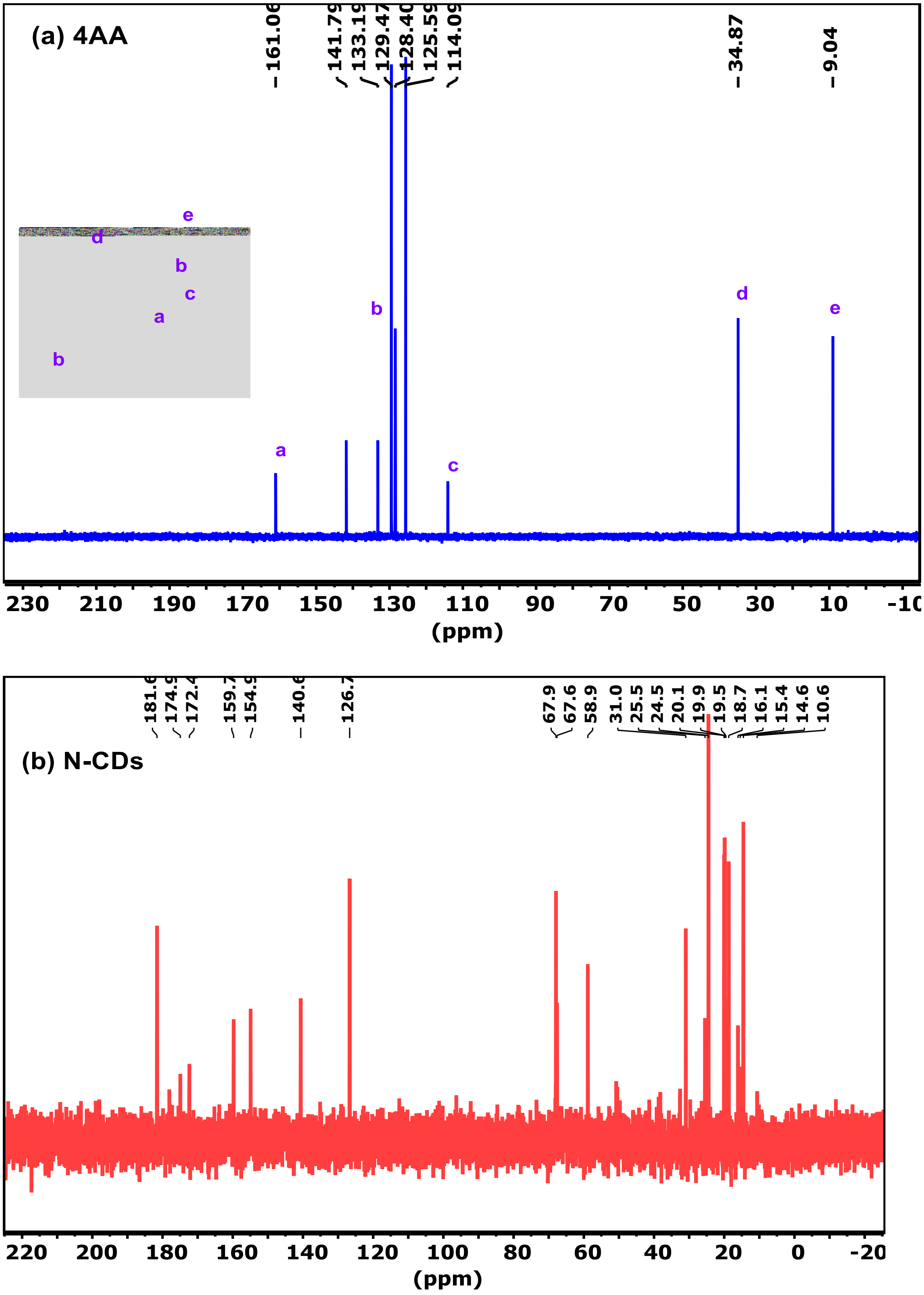

Fig. S3 ^{13}C-NMR spectra of **(a)** 4AA, and **(b)** N-CDs.

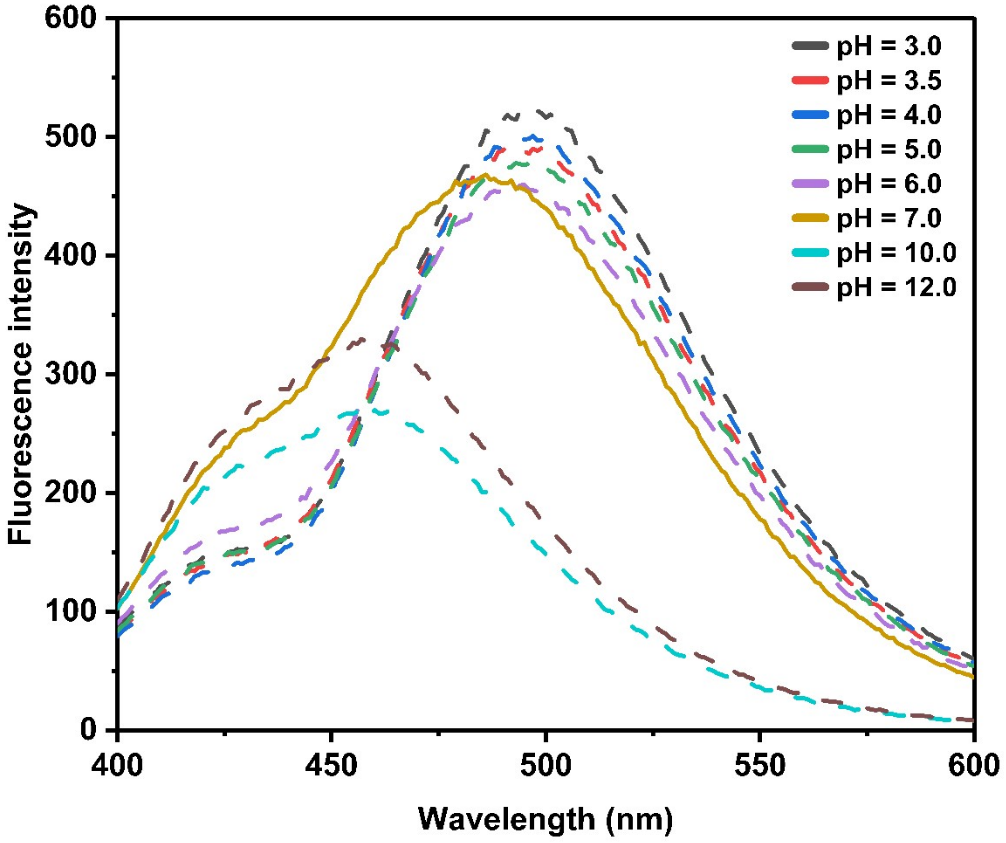

Fig. S4 The fluorescence intensity of N-CDs and LEV solutions at pH levels ranging from 3 to 12 with different buffer solutions.

5. Quantitative XPS Analysis

The quantification of functional groups on the surface of the N-CDs was carried out through XPS analysis. The elemental atomic percentages were first obtained from the wide survey spectrum using the corresponding relative sensitivity factors (RSFs) for each element. The corrected atomic percentage of a given element *i* was calculated according to:

$$Atomic\%\ of\ component\ (i) = \frac{\frac{Area(i)}{RSF(i)}}{\sum \frac{Area(j)}{RSF(j)}} \times 100$$

where Area(i) is the integrated peak area of the element i in the survey spectrum, and RSF(i) is its relative sensitivity factor. The denominator represents the sum of all corrected peak areas for the detected elements. The elemental composition of the N-CDs determined from the XPS survey spectrum is provided in **Table S1**.

Table S1. Elemental composition of the N-CDs obtained from XPS survey spectra.

Name	Peak Area (CPS. eV)	Sensitivity Factor (SF)	Atomic% after SF correction
**C 1s**	34341.7	1.000	67.35
**N 1s**	18079.1	1.676	23.21
**O 1s**	13386.3	2.881	9.45

These normalized atomic percentages were then used to deconvolute the high-resolution spectra (C 1s, N 1s, O 1s). The fractional contribution (f_i) of each chemical component within a given element was calculated as:

$$f_i = \frac{A_i^{HR}}{\sum A_j^{HR}}$$

where A_i^{HR} represents the background-corrected peak area of component *i*.

To express the abundance of functional groups as a percentage of the overall surface composition (as reported in Table 1 of the main article), the survey-level atomic percentage (At.%) was combined with the high-resolution fractions according to:

$Group\% \ (i) = At.\%(i) \ \times \ f_i$

Table. S2 Determination and recovery of LEV in three commercial LEV tablets using a ratiometric fluorescence method.

Tablet	Spiked (µM)	Average Found (µM)	Std. dv	Spike recovery (%)
Pioneer	0	0.029	0.123	
	4	4.050	0.198	100.51
	8	7.099	0.426	88.36
Mascot	0	-0.056	0.587	
	4	3.569	0.553	90.62
	8	6.908	0.463	87.06
Aristo	0	0.514	0.593	
	4	3.475	0.150	85.04
	8	7.346	0.471	85.40

Table. S3 Determination and recovery of LEV in three commercial LEV tablets using a smartphone-based platform method.

Tablet	Spiked (µM)	Average Found (µM)	Std. dv	Spike recovery (%)
Pioneer	0	-0.117	0.072	
	4	3.415	0.166	88.38
	8	7.325	0.259	93.06
Mascot	0	0.580	0.136	
	4	4.312	0.317	93.31
	8	7.347	0.436	84.58
Aristo	0	0.137	0.095	
	4	3.903	0.0718	94.07
	8	7.269	0.273	89.12
